# Inheritance, Fitness Cost, and Management of Lambda-Cyhalothrin Resistance in a Laboratory-Selected Strain of *Ceratitis capitata* (Wiedemann)

**DOI:** 10.3390/insects11090551

**Published:** 2020-08-19

**Authors:** Ana Guillem-Amat, Elena López-Errasquín, Lucas Sánchez, Miguel González-Guzmán, Félix Ortego

**Affiliations:** 1 Departamento de Biotecnología Microbiana y de Plantas, Centro de Investigaciones Biológicas Margarita Salas, CSIC, 28040 Madrid, Spain; aguillem@cib.csic.es (A.G.-A.); elena.lopez@cib.csic.es (E.L.-E.); mguzman@uji.es (M.G.-G.); 2 Departamento de Biología Celular y Molecular, Centro de Investigaciones Biológicas Margarita Salas, CSIC, 28040 Madrid, Spain; lsanchez@cib.csic.es

**Keywords:** pyrethroid, insecticide resistance, simulation studies, digestive physiology, spinosad

## Abstract

**Simple Summary:**

The Mediterranean fruit fly (medfly), *Ceratitis capitata*, is considered one of the most destructive and economically damaging pests of citrus and other fruit crops worldwide. Current control practices in Spain rely on the use of insecticides (mainly lambda-cyhalothrin, spinosad, and deltamethrin) and the release of sterile males. However, the sustainability of medfly control programs is threatened by reports of resistance to lambda-cyhalothrin in field populations. In this work, we used a laboratory-selected lambda-cyhalothrin-resistant strain to study key factors required for devising effective insecticide resistance management strategies. Specifically, we have (1) determined that the inheritance of resistance is autosomic (non-associated to the sexual chromosome), completely dominant (a single copy of the gene is enough to confer resistance), and polygenic (controlled by more than one gene); (2) observed that resistant individuals present fitness alterations in regard to biological parameters (lower survival in the first growth stages, a slower developmental time, and higher adults’ weight and longevity); and (3) shown under laboratory conditions that the alternation of lambda-cyhalothrin with spinosad helped delay the development of resistance. Taken together, our results indicate that it would be advisable to encourage the rotation of these insecticides to manage the resistance problem.

**Abstract:**

The management of the medfly, *Ceratitis capitata*, in Spanish citrus crops relies mainly on the use of insecticides and the release of sterile males. However, the development of resistance to different insecticides in field populations, including lambda-cyhalothrin, implies a threat for the sustainable control of this pest. The inheritance, fitness cost, and management of lambda-cyhalothrin resistance were examined in the laboratory-selected W-1Kλ strain. We have demonstrated that lambda-cyhalothrin resistance in W-1Kλ is autosomic, completely dominant, and polygenic. In addition, individuals from W-1Kλ showed a lower embryo to pupal viability, a slower developmental time from egg to pupae, and an increase in adults’ weight and longevity. We did not find significant trade-offs in the activity of digestive hydrolytic enzymes, with the exception of higher α-amylase activity in W-1Kλ females. A comparative study with different insecticide treatment strategies showed that lambda-cyhalothrin resistance increased when several consecutive treatments with this insecticide were applied. However, the alternation of this insecticide with spinosad was enough to delay the development of resistance. Our results indicate that the rotation of lambda-cyhalothrin with spinosad—a practice already used in some fields—may contribute to prevent the development of resistance.

## 1. Introduction

The Mediterranean fruit fly (medfly), *Ceratitis capitata* (Wiedemann), is one of the main insect pests of fruits. It is widespread in the Mediterranean basin, Africa, Middle East, and South America [1], where it causes remarkable economic damage to citrus and other fruit crops. Current control practices against medfly in citrus crops in Spain rely on the use of insecticides, mainly spinosad, lambda-cyhalothrin, and deltamethrin, and the release of sterile males. However, the sustainability of management programs and the durability of available insecticides are threatened by the evidence of field-evolved resistance to malathion [2] and lambda-cyhalothrin [3]. Moreover, although medfly susceptibility to spinosad remains, resistant alleles for this insecticide have recently been discovered in field populations [4]. In this context, the implementation of Insecticide Resistance Management (IRM) strategies aimed at either overcoming resistance to currently used compounds or preventing the development of resistance to extant or new insecticides becomes essential [5,6]. Besides, IRM strategies will contribute to keep the use of these pesticides only to levels that are economically and ecologically justified, as required by current regulations for a sustainable use of pesticides [7].

Knowledge about the different aspects that condition insecticide resistance appearance and evolution is needed for devising effective IRM strategies, and laboratory-selected strains are very useful for their study. Understanding the mechanism of resistance will provide some degree of intervention and management, such as the use of synergists in the case of metabolic resistance, the design of diagnostic tools to detect and track field resistance, and the use of pesticides with a different mode of action in the case of cross-resistance [5,8]. The way resistance is inherited determines how it would be transmitted to the offspring. Important aspects to be considered are to find out if the resistant traits are recessive or dominant, if they are autosomal or sex-linked, and if they are under monogenic or polygenic control [9]. The fitness cost linked to insecticide resistance also has an effect on the development and spread of resistant populations. The higher the fitness cost, the longer the time for resistant individuals to spread in the population, thus adding an important factor to take into consideration when developing resistance management programs [10].

The mechanism of resistance to lambda-cyhalothrin in medfly has been studied in the laboratory resistant strain W-1Kλ, which was obtained after selection with this insecticide. Reversion of resistance by the synergist piperonyl butoxide (PBO) and over-expression of the *CcCYP6A51* gene detected by qPCR pointed to a P450-mediated metabolic type of resistance [3]. Moreover, functional validation by the expression of this gene in *Escherichia coli* and in *Drosophila melanogaster* (Meigen) confirmed, respectively, its ability to metabolize pyrethroids and that its induced expression confers a resistant phenotype [11]. Nonetheless, it has been reported that the expression of *CcCYP6A51* presents a very heterogeneous pattern with high variability, both between and within field populations susceptible to pyrethroids from Greece [12]. Besides, we cannot discard that resistance arose as a combination of several genes with different patterns of expression. Therefore, the expression level of P450 genes is not a reliable method for resistance monitoring, and the absence of more accurate information involving the resistance mechanism has not allowed the design of a proper diagnostic tool for this type of resistance yet.

Previous studies in dipterans have found that the inheritance of resistance to lambda-cyhalothrin [13] and other pyrethroids [14,15] varies from dominant to recessive when mediated by P450s. Besides, a single gene or polygenic traits can be involved in metabolic resistance to pyrethroids, including different CYP genes [16] and other genes associated with pesticide metabolism [17,18]. Regarding the fitness cost of pyrethroids resistance in dipterans, it has been reported that this chemical family negatively affects the life traits of resistant strains, as extensively observed in *Aedes aegypti* (L.) [19,20,21] and in *Musca domestica* L. [22], in which resistance has also appeared to be unstable. Considering the metabolic mechanisms of pyrethroids resistance, it is known that if the over-expression of an enzyme associated with resistance is energetically costly, resistant individuals over-producing such an enzyme in the absence of the insecticide would be at an energetic disadvantage [23]. For instance, it has been reported that individuals of *Culex pipiens* L. with permethrin resistance mediated by P450 detoxification had lower energetic reserves (glycogen and lipids) than their susceptible counterparts [24]. In addition, metabolic resistance to pyrethroids may interfere in other metabolic functions, such as digestive physiology in *C. pipiens* [25]. However, the inheritance and fitness cost of resistance to lambda-cyhalothrin in *C. capitata* have not been determined yet.

IRM programs usually propose rotations with different insecticides, which implies temporal cycles that alternate the use of compounds with different mechanisms of action [26,27]. Medfly control in citrus crops in Spain relies on the repeated use of lambda-cyhalothrin and spinosad, and the simultaneous use of one of these two chemicals with lure-and-kill traps of deltamethrin. Remarkably, the lambda-cyhalothrin-resistant W-1Kλ strain showed high levels of cross-resistance to deltamethrin but not to spinosad [3], which suggests that it would be advisable for medfly control to rotate lambda-cyhalothrin and spinosad. In a previous study, a resistance evolutionary model based on empirical data on spinosad resistance in medfly (inheritance, fitness cost, and frequency of resistant alleles in the field) led us to conclude that rotations with spinosad and an insecticide from a different chemical family would help manage the development of resistance [4]. However, a similar approach to investigate how lambda-cyhalothrin resistance could evolve has not been explored, because most of the required parameters are unknown.

The aim of this work was to investigate in the laboratory-selected strain W-1Kλ, some key factors that may play a role in lambda-cyhalothrin resistance development. Specifically, we have (1) determined the inheritance of resistance to lambda-cyhalothrin in W-1Kλ, (2) assessed biological and physiological fitness traits associated to lambda-cyhalothrin resistance in W-1Kλ, and (3) generated a laboratory multi-resistant strain to study susceptibility variations when rotations with lambda-cyhalothrin and spinosad are performed.

## 2. Materials and Methods

### 2.1. Laboratory Strains of C. capitata

A susceptible laboratory strain of *C. capitata*, C, has been reared in the laboratory without any insecticide exposure since 2001, when individuals were collected from non-treated experimental fields at the Instituto Valenciano de Investigaciones Agrarias (IVIA, Valencia, Spain). W-1Kλ is a lambda-cyhalothrin-resistant strain, which was generated in the laboratory from the malathion-resistant W-4Km strain, by selection with increasing lambda-cyhalothrin concentrations for several generations [3]. The spinosad-resistant JW-100s strain was established from field individuals collected in Xàbia in 2007; it has been selected with this insecticide in the laboratory since then; and its resistance mechanism has been associated to mutations in the target site, the α6 subunit of the nicotinic acetylcholine receptor *Ccα6* [28].

### 2.2. Crosses for Inheritance Study

Adults from W-1Kλ and C strains were collected, and their sex was determined immediately after adult emergence to assure virginity. Males and females from each strain were placed separately into ventilated plastic dishes and maintained in an environmentally controlled chamber (Sanyo MLR-350-H, Sanyo, Japan), at 25 ± 1 °C and a 16 h light and 8 h dark photoperiod (standard conditions) for two days. Reciprocal crosses (120 ♂ W-1Kλ × 120 ♀ C and 120 ♀ W-1Kλ × 120 ♂ C) were performed to obtain the F1 generation (F1A and F1B, respectively). The offspring of both reciprocal crosses was pooled and kept in the absence of selection pressure to produce the F2 generation. F1 was also backcrossed to the susceptible parent strain, C ((30 ♂ F1A + 30 ♂ F1B) × 60 ♀ C and (30 ♀ F1A + 30♀ F1B) × 60 ♂ C), to obtain the backcross (BC henceforth).

The dominance value (D_LC_) of W-1Kλ resistance was calculated using Bourguet’s modification [29] of Stone’s formula [30], which results in a range 0–1: D_LC_ = {[(2 logLC_50_ F1–logLC_50_ P1–logLC_50_ P2)/(logLC_50_ P1–logLC_50_ P2)] + 1}/2; where P1 and P2 corresponded to parental strains W-1Kλ and C, respectively. Values ranged between 0 for completely recessive and 1 for completely dominant.

### 2.3. Generation of a Multi-Resistant Strain of C. capitata

**Figure 1:**
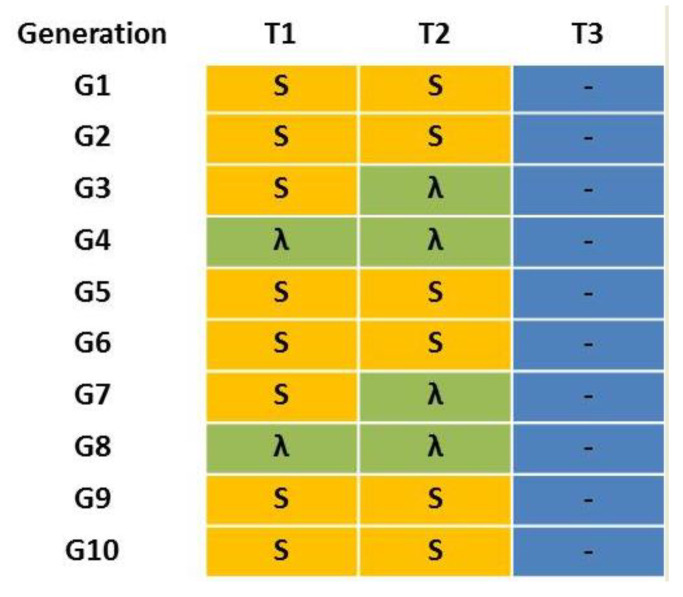
Treatment scenarios T1–T3 for the multi-resistant MR laboratory strain of *Ceratitis capitata*. “S” in yellow means a treatment with 100 ppm of spinosad, “λ” in green means a treatment with 125 ppm of lambda-cyhalothrin and “-” in blue means absence of insecticide treatment.

The laboratory strains W-1Kλ and JW-100s were crossed to generate a multi-resistant strain (MR). Adults from both strains were collected and their sex was determined immediately after adult emergence. Then, males and females from each strain were placed separately into ventilated plastic dishes and maintained with water and a rearing diet for two days. Reciprocal crosses (100 ♂ W-1Kλ × 100 ♀ JW-100 s and 100 ♀ W-1Kλ × 100 ♂ JW-100 s) were performed to obtain the F1 generation. The offspring of both reciprocal crosses was kept at standard conditions without insecticide exposure to produce the F2 generation. This was considered the initial MR strain for the experiment (G1), where all the possible allelic combinations were represented. At this point, the MR strain was divided into three sublines to emulate different treatment scenarios combining the most used insecticides at present in Spanish citrus crops: T1 alternated three consecutive generations with a treatment of 100 ppm of spinosad (an insecticide without cross-resistance to lambda-cyhalothrin) with a generation with 125 ppm of lambda-cyhalothrin treatment; T2 alternated a treatment of 100 ppm of spinosad in two consecutive generations with 125 ppm of lambda-cyhalothrin treatment during the next two generations; and T3 did not receive any insecticide treatment. The concentration used for lambda-cyhalothrin is the same as that applied in bait sprays in the field, whereas that for spinosad is lower than that used in the field (260 ppm) because of the limited solubility of spinosad in aqueous solutions. Treatments were applied during 10 consecutive generations (Figure 1).

### 2.4. Bioassays

Feeding bioassays were performed with lambda-cyhalothrin (KarateZeon 10% p/v (100 g/L), Syngenta Limited, Surrey, UK) and spinosad (Dow AgroSciences 88% p/p, Indianapolis, IN, USA). Adult flies 3–5 days old were tested. Five concentrations of each insecticide were tested in concentration–response assays. They were prepared by adding 1 mL of the diluted insecticide to 0.9 g of the rearing diet without water and homogenizing in a mortar. Dilutions of insecticides were prepared with water in the case of lambda-cyhalothrin, and with a buffer composed of acetic acid: sodium acetate (1:3, pH 4.7) in the case of spinosad. These solvents (water or buffer) were mixed with the rearing diet for the non-treated controls. Three replicates of 10–15 flies per replica were performed at each concentration. During the assays, flies were kept in ventilated plastic dishes (89 mm in diameter and 23 mm in height) at standard conditions. Flies were starved for 24 h before the exposure to insecticide. Mortality was recorded after 48 h and flies were considered dead if they were ataxic.

### 2.5. Assessment of Life History Traits

#### 2.5.1. Adults’ Weight and Longevity

Thirty females and 30 males (2–3 days old adult flies) of each strain were placed separately in ventilated plastic dishes, fed with water and rearing diet (4:1:0.1, glass sucrose/hydrolyzed yeast/water) and kept in an environmentally controlled chamber at a photoperiod of 16 h light and 8 h dark at 25 ± 1 °C to measure daily survival. Thirty females and 30 males were kept in the same way for 3 days and weighed with a precision balance (AM100, Mettler-Toledo, Zurich, Switzerland). Three replicates were performed for each population and parameter.

#### 2.5.2. Fecundity

Females 7–10 days old were randomly selected and placed in circular ventilated plastic boxes (5 × 11 cm diameter) with water and a rearing diet in an environmentally controlled room at a photoperiod of 16 h light and 8 h dark and a temperature of 26 ± 3 °C. The number of laid eggs was counted every 24 h over 3 days. Six replicates with 20 females of each strain were performed.

Lifetime fecundity was also analyzed placing 7–10-day-old adult flies of each strain in ventilated plastic boxes (20 × 20 × 20 cm). The boxes were kept as described before until the flies died, and the eggs were collected weekly and measured volumetrically. Four replicates with 30 females and 30 males of each strain were performed.

#### 2.5.3. Embryo to Pupal Viability and Developmental Time to Pupation

A volume of 50 μL of eggs (containing at least 500 eggs, estimated visually) of less of 24 h of age were collected and spread on larval rearing medium (160 g approximately) in containers (130 × 90 × 25 mm) covered with an aluminum foil to avoid desiccation. Containers were placed in 2 L ventilated plastic boxes kept in an environmentally controlled chamber at a photoperiod of 16 h light and 8 h dark at 25 ± 1 °C. Third instar larvae that jumped from the food container and pupated were daily recorded and removed from the box. Three replicates of each strain were performed.

### 2.6. Enzymatic Activities

Biochemical analyses were carried out to determine the specific activities of the main hydrolytic enzymes in medfly adults [31]. Adult flies 3–4 days old were collected and frozen in liquid nitrogen. Protein extracts were obtained by homogenizing the abdomen and thorax from single individuals in 200 μL of 0.15 M NaCl, centrifuging at 12,000 rpm for 5 min, and collecting the supernatant. Total protein content was determined according to the method of Bradford [32]. Twelve males and 12 females were analyzed per strain. Unless otherwise stated, all enzymatic activities were performed at their optimum pH of activity in 200 μL of reaction mixture. Blanks were used to account for the spontaneous breakdown of substrates, and all assays were done in duplicate.

Trypsin-like activity was measured using t-Butyloxycarbonyl-L-glutaminyl-glycyl-L-arginine-4-methylcoumaryl-7-amide (Boc-QGR-AMC) (PeptaNova GmbH, Sandhausen, Germany) as substrate. Samples of 10 μL of fly extracts were incubated with 20 μM Boc-QGR-AMC in 0.1 M Tris-HCl buffer (0.15 M NaCl, 5 mM MgCl_2_, pH 8.5) at 30 °C for 1 h. Fluorescence was measured using an excitation filter of 340 nm and an emission filter of 460 nm on a Varioskan Flash reader (ThermoFisher Scientific, Willington, CT, USA). A calibration curve was obtained with known amounts of 7-amino-4-methylcoumarin (AMC) (Bachem, Bubendorf, Swizerland).

Leucine aminopeptidase-like activity was determined using L-Leucine p-nitroanilide (LpNa) (Sigma, St Luis, MO, USA) as substrate. Samples of 20 μL of fly extracts were incubated with 1 mM LpNa in 0.1 M Tris-HCl buffer (0.15 M NaCl, 5 mM MgCl_2_, pH 7.0) at 30 °C for 1 h. The reaction was stopped with 100 μL of 30% acetic acid, and the absorbance was measured at 410 nm on a VERSAmax microplate reader (Molecular Devices Corp., Sunnyvale, CA, USA), using a molar extinction coefficient of 8800 M^−1^ cm^−1^ for pNa.

α-Amylase activity was measured using starch as substrate as described by Valencia [33]. Samples of 10 μL of fly extract were incubated with 90 μL of 0.1 M Tris-HCl buffer (40 mM CaCl_2_, 20 mM NaCl, pH 7.0) and 100 μL of 0.5% starch solution at 30 °C for 2 h. The reaction was stopped by adding 1 mL of lugol solution (0.02% I2, 0.2% KI). The mixture was centrifuged at 6000 rpm for 5 min, and the supernatant’s absorbance was measured at 580 nm on a VERSAmax microplate reader. A starch standard curve was used as a reference.

Cathepsin D-like activity was determined using (7-Methoxycoumarin-4-yl) acetyl-glycyl-L-lysyl-L-prolyl-L-isoleucyl-L-leucyl-L-phenylalanyl-L-phenylalanyl-L-arginyl-L-leucyl-[Nε–(2,4-dinitrophenyl)-L-lysyl]-D-arginine amide (MocAc-GKPILFFRLK(Dnp)-D-R-NH2) (PeptaNova GmbH, Sandhausen, Germany) as substrate. The assays were performed incubating 2.5 μL of the fly protein extract with 87.5 μL of 0.1 M citric acid–NaOH buffer (0.15 M NaCl, 5 mM MgCl_2_, pH 2.5) and 20 μM substrate at 30 °C for 15 min. Fluorescence was measured using an excitation filter of 328 nm and an emission filter of 393 nm on a Varioskan Flash reader, and MocAC-Pro-Leu-Gly (MCA) (Peptanova GmbH, Germany) was used as standard.

### 2.7. Statistics

Data were statistically analyzed with Levene and Shapiro–Wilk tests to check the homogeneity and normality, respectively. Susceptibility to lambda-cyhalothrin and the spinosad of *C. capitata* laboratory strains and crosses was analyzed using mortality data to estimate LC_50_ values (concentration needed to cause 50% mortality). Probit analysis was performed using the program POLO-PC (LeOra Software14, LeOra, Berkeley, CA, USA), which corrects samples’ mortality by control mortality using Abbott’s transformation [34]. Resistance ratios (RR = LC_50_ (lab strain)/LC_50_ (C strain)) were considered significant if their 95% fiducial limits did not include 1 [35]. In the inheritance study, a χ^2^ test was performed to prove the fit of the data to a monogenic model. In the biological cost analysis, data were analyzed by one-way ANOVA, followed by Tukey’s *post hoc* test for adults’ weight, daily and lifetime fecundity, number of pupae and developmental time from egg to pupa, and by the Kruskal-Wallis *post hoc* test corrected by Bonferroni for adults’ weight and enzymatic activities. Data were Ln(x + 1) transformed in the case of embryo to pupal viability. The Kaplan–Meier method was used to analyze adult survival, and their distributions were compared by the Mantel-Cox log-rank test.

## 3. Results

### 3.1. Inheritance of Lambda-Cyhalothrin Resistance

The inheritance pattern of lambda-cyhalothrin resistance in the laboratory-selected W-1Kλ strain was studied by performing crosses and analyzing the susceptibility to the insecticide of parental strains (resistant W-1Kλ and susceptible C) and F1, F2, and BC (Table 1). It was tested by feeding bioassays, since the ingestion of baits containing the insecticide is the way of exposure in the field. Mortality values below 15% were obtained with the W-1Kλ strain for concentrations up to 1000 ppm. The W-1Kλ strain could not be tested at concentrations over 1000 ppm because of the repellent effect of lambda-cyhalothrin on *C. capitata* at higher concentrations (personal observations). As we could not estimate the exact LC_50_ value for W-1Kλ, we assumed that it was over 1000 ppm; thus, the resistance ratio (RR) of W-1Kλ with respect to C (LC_50_ = 17 ppm) was estimated as > 59. The mortality values of F1 reciprocal crosses turned out to be equivalent to the resistant parent’s and equal in both reciprocal crosses, indicating that lambda-cyhalothrin resistance was completely dominant and autosomic. As before, we assumed that the LC_50_ values for F1 crosses were over 1000 ppm, which allows us to estimate the RR values as > 59 and the dominance values (D_LC_) as close to 1. The RR values for the F2 and BC crosses were 12.0 and 3.7, respectively. In both cases, the observed mortality at the discriminating concentration of 300 ppm (96% mortality for susceptible parental C and 3% mortality for resistant parental W-1Kλ) did not fit the mortality expected for a dominant character under a monogenic model, as we observed 65% in the F2 and 84% in the BC, while the expected values would have been 25% and 50%, respectively (Table 1). It means that the inheritance of lambda-cyhalothrin resistance in the W-1Kλ strain is not controlled by a single gene with Mendelian genetics.

### 3.2. Fitness Cost Associated to Lambda-Cyhalothrin Resistance in W-1Kλ

Biological and physiological parameters were evaluated to determine if individuals of the lambda-cyhalothrin-resistant W-1Kλ strain present a fitness cost when compared to individuals of the susceptible C strain and of the malathion-resistant W-4Km strain from which W-1Kλ was generated.

**Figure 2:**
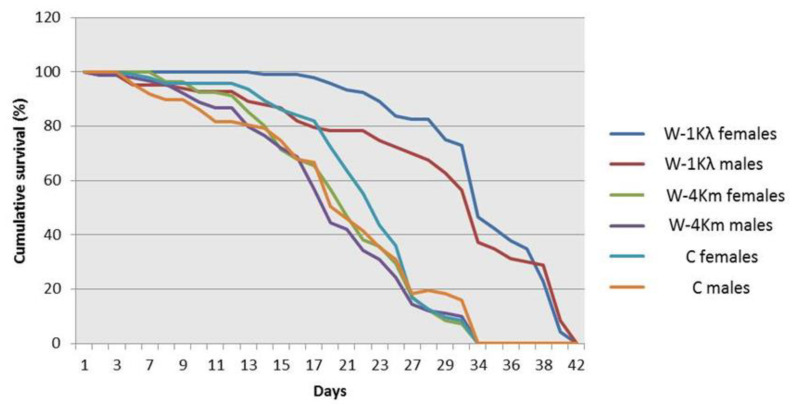
Adult longevity of males and females from the lambda-cyhalothrin-resistant W-1Kλ strain, the susceptible C strain, and the malathion-resistant W-4Km strain of *Ceratitis capitata*. Kaplan–Meier survival curves were compared using the Log-Rank test (Mantel–Cox) at *p* ≤ 0.05. Different letters mean significant differences.

Our results indicate that individuals from W-1Kλ showed a reduction in embryo to pupal viability and a slower developmental time from egg to pupa when compared to individuals from the C and W-4Km strains (Table 2). However, both adult males and females from W-1Kλ were significantly heavier (Table 2) and their longevity was longer (Figure 2) than those from the C and W-4Km strains. To compare fecundities, the number of eggs laid by 20 7–10-day-old females was recorded every day for 3 days (Table 2). Different daily trends were observed, but when the data of the 3 days were pooled, egg laying in W-1Kλ was significantly lower than in the susceptible C strain, but not when compared to W-4Km. In addition, since the longevity of W-1Kλ was longer, we also checked the lifetime fecundity by weekly recollecting the eggs laid by 30 females until the flies died to measure the total final eggs volume. We found that the volume of eggs was significantly lower in W-1Kλ than in the C strain, but not when compared to W-4Km.

Biochemical analyses were also carried out to determine the specific activities of hydrolytic enzymes that may indicate alterations in medfly digestive physiology (Table 3). Proteolytic digestion in medfly relies on aspartyl and serine proteases and exopeptidases [31], whereas α-amylases are involved in the hydrolysis of dietary carbohydrates [36]. No significant differences between adult males and females within each strain were observed for any of these activities. When strains were compared, W-1Kλ males presented higher α-amylase activity and lower leucine aminopeptidase-like activity than C males, whereas W-1Kλ females showed higher α-amylase activity than C and W-4Km females. The only difference between the W-4Km and C strains was that α-amylase activity was higher in W-4Km males. No significant differences among strains were observed for total protein and trypsin-like and cathepsin D-like activities.

### 3.3. Impact of Different Treatment Scenarios in the Progression of Resistance in the Multi-Resistant Laboratory Strain MR of C. capitata

We generated a multi-resistant strain (MR) by reciprocal crosses of JW-100s (resistant to spinosad) and W-1Kλ (resistant to lambda-cyhalothrin) strains and keeping the offspring for two generations without exposure to insecticides. At this point (G1), we established three scenarios of insecticide treatments to represent rotations between lambda-cyhalothrin and spinosad, which are the most used insecticides at present in Spanish citrus crops (Figure 1). We observed how lambda-cyhalothrin and spinosad resistance evolved along the generations depending on the treatments received.

The results for lambda-cyhalothrin can be observed in Table 4. The initial situation (G1) showed a very heterogeneous response in the susceptibility of the MR strain to this insecticide. The variability in individuals’ response, manifested by a low slope and high fiducial limits (LC_50_ = 50.63 (3.89–129.87 ppm)), was expected considering that (1) resistance to lambda-cyhalothrin is controlled by more than one gene according to our inheritance results; and (2) all genotypes can be potentially represented in G1 (corresponding to a typical F2 cross). In MR-T1, LC_50_ values increased after the lambda-cyhalothrin treatment (G5 and G9), while they diminished again after two generations with spinosad treatment (G7 and G11), an insecticide for which lambda-cyhalothrin has no cross-resistance. Thus, although we proved that lambda-cyhalothrin resistance evolved fast in the presence of selection pressure, a generation of relaxation of this pressure was enough to go backwards in the evolution of resistance. In MR-T2, we found the highest LC_50_ values in G5 and G9 (although they were not significantly different in this generation, which was probably due to the high fiducial limits), after two generations of lambda-cyhalothrin treatments. Interestingly, these values were higher than those found in MR-T1 (G5 and G7), where only a generation with lambda-cyhalothrin exposure was introduced between the spinosad treatments, suggesting that several consecutive treatments with lambda-cyhalothrin have a powerful effect on the selection of resistance. The relaxation of selection pressure was noticeable in G7 and G11, after two generations with spinosad treatments. MR-T3 (non-treated) showed random changes in susceptibility accompanied by large fiducial limits, which could mean that some individuals from this strain still kept the resistance mechanisms from the parental W-1Kλ and were able to escape the insecticide action when tested in the bioassays. In regard to spinosad resistance, results can be observed in Table 5. In MR-T1 and MR-T2, the first treatment was done with spinosad, and resistance was fixed since this moment. Due to this, mortality values when exposed to 100 ppm of spinosad were close to 0 in all the generations analyzed. In MR-T3 (non-treated), susceptibility increased since G1 along the experiment. It is due to the absence of spinosad treatments that allowed the enrichment of the strain in susceptible alleles.

## 4. Discussion

In this work, we have demonstrated that resistance of the laboratory W-1Kλ strain to lambda-cyhalothrin is completely dominant, autosomic, and does not fit a monogenic model. Pyrethroids resistance caused by alteration of the target site, the voltage-gated sodium channel (VGSC), usually has an incompletely recessive inheritance pattern [37,38]. On the contrary, the case of metabolic resistance is variable. Examples go from recessive inheritance, as observed in *Culex quinquefasciatus* Say for permethrin resistance [14], to dominant, as lambda-cyhalothrin resistance in *M. domestica* [13]. The number of genes involved in resistance also varies in concordance with the resistance mechanism. Pyrethroids resistance occasioned by a modification of the *VGSC* gene is usually monogenic [39], while metabolic resistance can be controlled by one [40] or several [13,15] genes. Lambda-cyhalothrin resistance in the laboratory-selected W-1Kλ strain had previously been associated with the over-expression of the *CcCYP6A51* gene [3,11]. The results obtained for the inheritance of lambda-cyhalothrin resistance in W-1Kλ fits with what is expected for a metabolic type of resistance, although this indicates that other polygenic traits must also be involved.

The results of this work show that resistance to lambda-cyhalothrin in W-1Kλ is associated with fitness alterations in regard to biological parameters. Actually, the W-1Kλ strain showed a reduction in fecundity and embryo to pupal viability, a slower developmental time from egg to pupa, and an increase in adults’ weight and longevity. Remarkably, these biological parameters, except fecundity, were altered with respect to both the susceptible C strain and the malathion-resistant W-4Km strain from which W-1Kλ was derived. Thus, most of the alterations in the fitness traits analyzed might be associated to the previously proposed metabolic type of resistance in W-1Kλ [3], since resistance to malathion in W-4Km is mainly linked to target site alterations [41]. It has been suggested that metabolic resistance may require the reallocation of energy resources, reducing the energy available for basic biological functions and generating trade-offs between insecticide resistance and key life history traits [23]. Previous works reported that metabolic resistance to pyrethroids has a negative effect on the biological traits on resistant strains of dipterans as *M. domestica* [22] and *A. aegypti* [19,20,42]. In addition, metabolic resistance may deplete the energetic stores of insects [24] and interfere in other metabolic functions, such as the digestive physiology [25,43]. However, with the exception of higher α-amylase activity in W-1Kλ females, we have not found significant differences in the hydrolytic enzymes of W-1Kλ and W-4Km strains that may indicate alterations in the fly digestive physiology. Some differences were observed between both W-1Kλ and W-4Km with respect to the C strain, which may be part of a non-specific insecticide-induced stress response or reflect genetic differences because of their different origins (W-1Kλ derived from W-4Km, whereas C was obtained from a different field population).

The simulation of lambda-cyhalothrin and spinosad resistance evolution when rotations of both insecticides are performed resulted in successful resistance management. The susceptibility of the MR strain to lambda-cyhalothrin decreased after each treatment with this insecticide, as expected by the completely dominant nature of resistance. Nonetheless, susceptibility to lambda-cyhalothrin sharply increased when treating with spinosad because (1) recessive susceptible alleles remain in the populations carried by heterozygotes, and (2) the alterations observed for some biological traits in lambda-cyhalothrin-resistant individuals may convey a fitness cost that contributes to the instability of lambda-cyhalothrin resistance. In the case of spinosad, a predictive model developed by our group aimed at the intensive and sole use of this insecticide could result in an increase in the frequency of spinosad-resistant alleles [4]. Although our simulation study started with a higher frequency of spinosad-resistant alleles than the above-mentioned model, it reinforced the idea that even though the recessive nature of spinosad resistance makes its evolution slow at the beginning, the spread of resistance can be very fast when resistant individuals are abundant in the population. Indeed, the difficulties to revert resistance when it is much expanded and treatments remain are also observed in our results. These results highlight the importance of combining the use of insecticides with different modes of action.

## 5. Conclusions

The findings of this work show that resistance to lambda-cyhalothrin in the laboratory-selected strain W-1Kλ has an autosomic, completely dominant pattern of inheritance, and that it is controlled by more than one gene. In addition, the alteration of biological parameters in this laboratory strain indicated that a fitness cost might be associated to the resistant phenotype. In this work, we have shown under laboratory conditions that the rotation of lambda-cyhalothrin with an insecticide with a different mechanism of action as spinosad, a practice already used in some fields, may contribute to prevent the development of resistance. However, care should be taken when extrapolating laboratory results to field populations because their environmental conditions and genetic backgrounds are not always comparable [23,29].

## Figures and Tables

**Table 1:** Study of the inheritance of resistance to lambda-cyhalothrin in the W-1Kλ strain of *Ceratitis capitata*.

Mortality (%) ± SE (n^1^) to Lambda-Cyhalothrin (ppm)							
Generation	3	10	30	100	300	1000	Non-Treated
Parents							
C	2 ± 2 (60)	35 ± 12 (58)	84 ± 4 (57)	98 ± 2 (59)	96 ± 2 (55)		16 ± 8 (55)
W-1Kλ					3 ± 2 (60)	14 ± 5 (59)	0 (60)
F1-crosses							
F1A (♂W-1Kλ × ♀C)		0 (30)	0 (30)	0 (30)	0 (30)	30 ± 12 (30)	0 (30)
F1B (♀W-1Kλ × ♂C)		0 (30)	0 (30)	0 (30)	3 ± 3 (40)	27 ± 3 (30)	3 ± 3 (30)
F2-crosses							
(F1A and F1B interbred)		10 ± 6 (60)	22 ± 11 (60)	42 ± 5 (60)	65 ± 10 (60)	77 ± 4 (60)	8 ± 6 (60)
Backcrosses							
BC^2^		2 ± 2 (59)	42 ± 6 (60)	73 ± 3 (60)	84 ± 3 (120)	92 ± 4 (60)	7 ± 5 (59)
Estimation of LC_50_, resistance ratio (RR), and dominance value (D_LC_)							
Generation	n^3^	Slope ± SE	LC_50_^4^ (95% FL)	χ^2^	df	RR^5^ (95% FL)	D_LC_^6^
Parents							
C	344	2.34 ± 0.24	17 (7−35)	116.4 *	18	1	
W-1Kλ	239		>1000			>59	
F1-crosses							
F1A (♂W-1Kλ × ♀C)	180		>1000			>59	~1
F1B (♀W-1Kλ × ♂C)	190		>1000			>59	~1
F2-crosses							
(F1A and F1B interbred)	360	1.41 ± 0.18	205 (115−348)	35.0 *	18	12.0 (7.4−19.4) ^#^	
Backcrosses							
BC^2^	418	1.57 ± 0.18	63 (38−98)	38.0 *	18	3.7 (2.4−5.7) ^#^	
Fit of the inheritance segregation to a monogenic model							
Generation	Mortality observed^7^		Mortality expected^8^		χ^2^		df
F2	39/60 (65%)		25%		51.2 ^+^		1
BC	101/120 (84%)		50%		56.0 ^+^		1

^1^ Number of flies tested (3–8 replicates of 10–15 flies each); ^2^ BC was constituted by ((♂ F1A and ♂ F1B) × ♀C) and ((♀F1A and ♀F1B) x ♂C); ^3^ Number of flies considered in the Probit analysis (including non-treated); ^4^ Lethal concentration (LC50) expressed in ppm of lambda-cyhalothrin in the diet; ^5^ Resistance ratio (RR) = LC50 (selected lab strain or cross)/LC50 (C strain). The fiducial limits for RR were calculated according to Robertson and Preisler [35]; ^6^ Dominance value (DLC) calculated following the formula DLC = {[(2 logLC_50_F1–logLC_50_P1–logLC50P2)/(logLC50P1–logLC50P2)] + 1}/2; where P1 and P2 corresponded to parental strains W-1Kλ and C, respectively [29,30]; ^7^ Number and percentage of observed dead individuals in the inheritance experiment when exposed to 300 ppm of lambda-cyhalothrin in the diet; ^8^ Percentage of expected dead individuals in a monogenic model; * Do not fit to the probit model (*p* ≤ 0.05); ^+^ Do not fit to the monogenic model (*p* ≤ 0.05); ^#^ RR is significant (*p* ≤ 0.05) if the 95% FL does not include 1.

**Table 2:** Biological parameters of flies from lambda-cyhalothrin-resistant W-1Kλ, the susceptible C, and the malathion-resistant W-4Km strains of *Ceratitis capitata*.

Biological Parameters	C	W-1Kλ	W-4Km
Adults’ weight (g)			
Males	6.24 ± 0.060 ^a^	8.37 ± 0.090 ^b^	6.94 ± 0.087 ^c^
Females	7.52 ± 0.062 ^a^	9.61 ± 0.089 ^b^	7.93 ± 0.012 ^c^
Fecundity			
Daily (Nᵒ eggs/20 females) Day 1	515 ± 37 ^a,b^	372 ± 52 ^a^	598 ± 24 ^b^
Day 2	765 ± 23 ^a^	645 ± 26 ^b^	551 ± 34 ^b^
Day 3	716 ± 20 ^a^	661 ± 43 ^a^	617 ± 35 ^a^
Days 1–3	1996 ± 48 ^a^	1678 ± 93 ^b^	1767 ± 41 ^a,b^
Lifetime (cm^3^ eggs/30 females)	1.50 ± 0.18 ^a^	0.98 ± 0.08 ^b^	1.10 ± 0.12 ^ab^
Embryo to pupal viability (Number of pupae)	772 ± 38 ^a^	278 ± 56 ^b^	537 ± 63 ^c^
Developmental time (Days from egg to pupae)	8.54 ± 0.15 ^a^	9.63 ± 0.16 ^b^	9.04 ± 0.02 ^a^

Data are mean ± standard error. Different letters within each row indicate significant differences (ANOVA, Tukey *post hoc* test for fecundity, embryo to pupal viability and developmental time, and Kruskal–Wallis for weight, at *p* ≤ 0.05).

**Table 3:** Total protein and digestive enzymatic activities of adult male and female flies from lambda-cyhalothrin-resistant W-1Kλ, the susceptible C, and the malathion-resistant W-4Km strains of *C. capitata*.

Enzymatic Activity	C		W-1Kλ		W-4Km	
	Males	Females	Males	Females	Males	Females
Total protein	341 ± 18 ^a,b^	361 ± 38 ^a,b^	285 ± 20 ^a^	381 ± 43 ^a,b^	321 ± 35 ^a,b^	433 ± 46 ^b^
Trypsin	0.42 ± 0.07 ^a^	0.46 ± 0.1 ^a^	0.41 ± 0.13 ^a^	0.38 ± 0.06 ^a^	0.26 ± 0.06 ^a^	0.19 ± 0.03 ^a^
Cathepsin D	17.1 ± 0.7 ^a,b^	17.7 ± 1.7 ^a,b^	21.2 ± 2 ^a^	18 ± 2.7 ^a,b^	18.5 ± 1.5 ^a,b^	13.3 ± 1 ^b^
Leucine aminopeptidase	28.7 ± 3.7 ^a^	29.1 ± 5.4 ^a,b^	12.5 ± 1.3 ^b^	12.8 ± 1.1 ^a,b^	13.5 ± 1.6 ^a,b^	11.9 ± 1.6 ^b^
α-Amylase	69.4 ± 4.2 ^a^	70.6 ± 8.7 ^a^	178.6 ± 23 ^c^	139.1 ± 11 ^c^	123 ± 11 ^b,c^	80.6 ± 8.1 ^a,b^

Data are mean ± standard error. Total protein data are expressed as µg per insect, and enzymatic activities as nmol of substrate hydrolyzed per min and mg of insect total protein. Different letters within each row indicate significant differences (Kruskal–Wallis test at *p* ≤ 0.05, *p* values corrected by Bonferroni).

**Table 4:** Susceptibility to lambda-cyhalothrin in the multi-resistant strain MR of *Ceratitis capitata* under three different scenarios of insecticide treatments, T1: alternation of spinosad for three generations and lambda-cyhalothrin for one generation; T2: alternation of spinosad for two generations and lambda-cyhalothrin for two generations; T3: non-treated. See Figure 1 for more details.

Scenario	Generation	N ^†^	Slope ± SE	LC_50_^‡^	95% FL	χ^2^	df	Significance	RR ^§^	95% FL	Significance
Initial MR	G1	285	0.80 ± 0.15	50.63	3.89–129.87	36.63	13		1		
T1	G5	179	1.71 ± 0.25	175.87	124.96–245.44	7.38	10	*	3.47	1.20–10.05	#
	G7	180	1.60 ± 0.38	27.59	11.52–41.30	7.17	10	*	0.54	0.16–1.82	
	G9	199	2.06 ± 0.35	233.76	168.85–345.03	15.23	13	*	4.62	1.63–13.10	#
	G11	225	1.06 ± 0.34	21.78	0.30–62.90	7.55	10	*	0.43	0.02–8.66	
T2	G5	149	1.87 ± 0.29	301.06	173.08–579.44	18.68	10		5.95	2.02–17.54	#
	G7	262	2.07 ± 0.31	130.70	91.10–209.64	35.05	15		2.58	0.93–7.19	
	G9	250	1.69 ± 0.23	272.79	159.31–460.66	48.07	18		5.39	0.01–1947.02	
	G11	224	1.03 ± 0.34	21.97	0.19–65.11	8.59	10	*	0.43	0.02–11.11	
T3	G5	181	1.81 ± 0.32	172.74	110.12–334.75	13.22	10	*	3.41	1.14–10.22	#
	G7	212	1.52 ± 0.26	23.05	8.08–39.78	27.21	13		0.46	0.14–1.41	
	G9	120	3.38 ± 0.61	32.38	22.55–47.18	10.44	7	*	0.64	0.23–1.82	
	G11	300	1.25 ± 0.20	246.94	128.7–913.9	37.89	14		4.88	1.55–15.32	#

^†^ Number of flies considered in the Probit analysis (including non-treated); ^‡^ Lethal concentration (LC_50_) in ppm of lambda-cyhalothrin in the diet for feeding bioassays at 48 h; ^§^ Resistance ratio (RR) = LC_50_ (selected strain)/LC_50_ (MR-G1 strain). The fiducial limits for RR were calculated according to Robertson and Preisler [35]; * Good fit of the data to the probit model (*p* > 0.05); ^#^ RR is significant (*p* ≤ 0.05) if the 95% FL does not include 1.

**Table 5:** Susceptibility to spinosad of the multi-resistant strain MR of *Ceratitis capitata* under three different scenarios of insecticide treatments, T1: alternation of spinosad for three generations and lambda-cyhalothrin for one generation; T2: alternation of spinosad for two generations and lambda-cyhalothrin for two generations; T3: non-treated. See Figure 1 for more details.

Scenario	Generation	Mortality % ± SE (n) ^†^				
		Non-treated	0.5 ppm	1 ppm	10 ppm	100 ppm
Initial MR	G1	2 ± 2 (45)	-	56 ± 11 (45)	71 ± 6 (45)	82 ± 4 (44)
T1	G5	0 ± 0 (36)	0 ± 0 (24)	0 ± 0 (36)	0 (12)	0 ± 0 (36)
	G7	0 ± 0 (36)	-	-	-	0 ± 0 (36)
	G9	0 ± 0 (30)	-	-	-	3 ± 3 (30)
	G11	2 ± 2 (45)	-	-	-	0 ± 0 (45)
T2	G5	0 ± 0 (29)	0 ± 0 (20)	0 ± 0 (30)	0 (10)	0 ± 0 (30)
	G7	0 ± 0 (36)	-	-	-	3 ± 3 (36)
	G9	0 ± 0 (30)	-	-	-	3 ± 3 (30)
	G11	2 ± 2 (45)	-	-	-	0 ± 0 (45)
T3	G5	3 ± 3 (36)	8 ± 8 (24)	67 ± 25 (36)	100 (12)	97 ± 3 (36)
	G7	6 ± 3 (36)	77 ± 3 (35)	89 ± 3 (36)	97 ± 3 (36)	94 ± 6 (36)
	G9	3 ± 3 (30)	53 ± 3 (30)	84 ± 3 (31)	97 ± 3 (30)	93 ± 3 (30)
	G11	0 ± 0 (45)	69 ± 4 (45)	98 ± 2 (45)	98 ± 2 (45)	100 ± 0 (45)

^†^ Flies tested (1–4 replicates of 10–15 flies each).
